# Contributions to Practical Surgery

**Published:** 1867-10

**Authors:** John Mettauer

**Affiliations:** Prince Edward C.H., Virginia


### Selections.

### CONTRIBUTIONS TO PRACTICAL SURGERY.

By JOHN METTAUER, M.D., LL.D., Prince Edward C.H., Virginia.

*Intrastructural Treatment of Tumors.*

Some twelve or fifteen years since, the writer treated an inteteting case of scrofula, in which the parotid, submaxillary, and sublingual glands of both sides were extensively implicated. The subject was a youth, about 9 or 10 years of age, well grown but pale, rather feeble, short-winded, and disinclined to take exercise. For a year before the case came into my hands, the disease had been slowly advancing, but had not been subjected to any regular treatment, as was stated by a senior member of the family, domestic remedies being chiefly employed. For a number of years, the writer has relied chiefly upon emetics and brisk purgatives, with restrictions on diet, and an equable state of the body temperature, in treating scrofula, more especially the form now under consideration; and a vast number of the cases yielded to such treatment, as was evidenced by the perfect cures following from it. In the case now under consideration, that plan, however, failed, after a fair trial; and the writer, believing any further attempt to medicate it through the constitution chiefly would endanger the life of the patient, determined to subject it to topical treatment by injecting into the bodies of the tumors some sorbefacient liquid; and the remedy selected was the tincture of iodine. The tincture employed was formed by dissolving half an ounce of iodine in eight ounces of alcohol, and from thirty to forty drops were injected into the tumors once in three or four days, using for the purpose a small glass syringe and a delicate trocar formed for the purpose. The central portions of the tumors were first injected, and in succession the remedy, from time to time, was applied to nearly every portion of them. Very slight pain attended and followed the operations, and the patient, comparatively a child, submitted to them readily, only complaining of a burning sensation in the tumors at the time of injecting them, and inconsiderable soreness afterwards.

This treatment was kept up for two months, at the expiration of which time the tumors had entirely disappeared, as well as the disgusting deformity caused by them, and the skin of the respective regions occupied by them appeared in all respects healthy, as well as smooth and comely. During its progress, the first perceptible changes in the condition of the tumors were, increased bulk and sensibility, and very slight exaltation of temperature. In ten days after the first injection was used, the tumors became somewhat lobulated, as well as softened under the taxis; and these changes progressively increased until they disappeared entirely.

To aid the sorbefacient operation of the intrastructural treatment, aperients were employed, and as the amelioration progressed, with the design of correcting anæmia, iodide of iron was superadded, after the tumors had become reduced more than two-thirds of their original bulk. During the treatment it could not be perceived that the constitution experienced any marked change, and certainly nothing like iodism made its appearance.

This was, unquestionably, an example of struma of well-marked character, and the subsidence of the tumors under the treatment to which they were subjected, and their final disappearance, were equally striking. There was evidently a decided constitutional aptitude for the disease, as the subject presented most of the appearances which indicate such a state, and was descended from scrofulous parents.

Some years after this case was treated, an example of fibro-adipose tumor was placed under my professional care, which, by reason of its size and situation on the neck, I determined to treat with intrastructural injections. This tumor was as large as a fist, completely filling the space between the right half of the inferior maxillary bone and corresponding clavicle, and was of soft, compressible character. Before coming into my hands, an extensive incision had been made into it, and from the information given me by the patient, the surgeon who made the incision represented that the tumor consisted of adipose and very loose fibrous matter, the latter greatly preponderating. So large and illy defined was the tumor, that I felt unwilling to attempt its extirpation with the knife, to say nothing of my reluctance to subject the patient, a young female about 16 years old, to a painful operation, which would necessarily be followed by an extensive and deforming cicatrix should recovery take place.

This patient evidently had a strumous aptitude, but less strongly marked than in the case above referred to; and although pale and anæmic in some degree, seemed to enjoy good general health. She informed me that the tumor had existed eight months, and that its development from the first discovery of it had been quite rapid, but almost entirely painless. Even when handled roughly, it seemed entirely free from tenderness, and imparted to the taxis a feeling of soft wool.

The injection employed in this case was the tincture of iodine, and of the strength as well as after the manner of that used in the above reported case. Three injections, after intervals of ten and twelve days, were introduced, which, at the expiration of six weeks, were followed by the perfect dispersion and removal of the tumor. The injections caused sufficient irritation to render the tumor hard, quite sensitive when handled, and to cause some pain in mastication or other motions of the lower jaw; but these conditions soon disappeared, and without being attended with, or succeeded by, suppuration. Very slight constitutional disturbance followed the injections, after the first; this caused a feverish state in moderate degree, attended with headache and loss of appetite of one or two days' duration. The injection was applied to the central portions of the tumor first, and then to the peripheral, a single time. As soon as the inflammatory hardness caused by the first central injection abated, it could be perceived that there was manifest diminution in the size of the tumor, which was the case after each application of the remedy. Some months after the disappearance of the original tumor, there was a slight return, or apparent return, of it; but regarding this as, perhaps, only accidental hypertrophy of the structures lately the seat of the disease, due chiefly to morbid impressibility following the treatment, and likely to subside after a short time spontaneously, nothing was done for it; and as the patient has not returned, after promising to do so should the tumor continue, it is probable she is again well.

At the date of this paper, the writer is treating an unpromising case of scrofulous tumefaction of the female mamma by intrastructural injection of the diseased organ. The mamma is extensively diseased, the entire gland seeming to be implicated and greatly enlarged, as well as indurated, painful and sensitive to the slightest touch. Besides the disease of the mamma, numerous other local manifestations of constitutional scrofula exist, yet the general health has not materially deteriorated, notwithstanding the patient's severe suffering and loss of sleep for several months. Four injections, thus far, have only been employed—three of tincture of iodine, and one of strong, pure acetic acid. The injections were applied first to the central portions of the mamma, and next to the peripheral, after intervals of ten and fifteen days. In this case, the remedy caused considerable pain, but comparatively very little inflammation of the parts injected, and no great constitutional disturbance, except sleeplessness; and after the trial of the remedy four times, it could not be perceived that any marked change had taken place in the diseased mamma, favorable or otherwise. The case is still under treatment, and should a fortunate result follow, which it is feared is quite improbable, it will surely be reported.

The writer thinks he derived the idea of the intrastructural treatment of tumors from the employment of firm compression, the seton, and acupuncture in such cases years since, in which successful results followed, and were no doubt due to the irritation set up by those agencies in the diseased parts, of a different character from that upon which such growths depend. That injecting an abnormal growth with either of the substances which have been named tends to set up in them irritation, and essentially to change the innervation as well as their nutritive action also, will not be denied; and firm compression, the seton, and acupuncture, it seems, must act chiefly in the same manner. Besides the contra-irritant action of the tincture of iodine, however, sorbefacient influences seem to be exerted by it likewise. Even acetic acid may act in like manner, though Dr. Broadbent, of London, thinks the injection of it into cancerous tissue arrests the growth, or causes the reäbsorption of it, by a solvent power its supposed by him to exert upon the living textures. It will not be profitable to attempt an explanation of the *rationale* of the discutient action of intrastructural injections in these cases, as it would be pure speculation; but it may be assumed, without fear of contradiction, that a change of vital action of the affected parts, both of the tumors and of the supporting textures, is the result of such treatment, a change which opposes the continuance as well as the further extension of them.

For many years, the writer has entertained the belief that the scrofulous diathesis, in whatever condition of the living organism it may consist, constitutes the true foundation of cancerous development. In every case of that disease committed to his care, and their number has been great, the disease was clearly connected with a strumous constitution more or less strongly marked. Frequently, too, scrofulous tumors of the glands have, after their long continuance, degenerated into cancer; and the removal of a cancerous parotid and mamma has as often been succeeded by phthisis, which, very probably, is scrofula of the lungs, or the phthisical form of cancer. There is no doubt that phthisis often precedes and follows the evolution of cancer in the same individual; and, *vice versa*, that the same is the case with scrofula and cancer of the mamma with respect to phthisis. Indeed, these cases seem to depend upon the same kind of morbid action; but the products differ, in some slight degree, perhaps, by reason of the higher or lower grade of phlogosis by which they are accompanied. And, if the method of treating scrofulous tumors by intrastructural injection is safe and beneficial, it would seem that it might prove equally so in cancerous growths likewise. The relationship of cancer and tubercle has been referred to by Dr. Thomas Weeden Cooke, in a work recently published in London, entitled, "Cancer, its Allies and Counterfeits;" and the writer of this paper is really gratified at finding his views on this subject, so long entertained, supported by such respectable authority. Dr. Broadbent, of St. Mary's Hospital, London, already referred to, proposes to treat cancer by injecting the diseased texture with acetic acid, and the results, in the cases thus treated, have been quite favorable. This method has also been tried in the Massachusetts General Hospital. It is exceedingly probable that it will be found as useful in the treatment of non-malignant as in scrofulous and cancerous tumors, with the exception, perhaps, of the bones when invaded by those diseases, and the second case referred to in this paper justifies such a belief.

### *Retention of Urine from Weakness of the Bladder, cured by Injections of Tea.*

Some years since, the writer was called, in consultation, into a neighboring State, to see a highly respectable gentleman, in a case of retention of urine. The gentleman had been laboring under the disease some eight or nine months, was about 55 or

60 years of age; previous to his attack, was of vigorous general health, of active habits, and, as is too common with wealthy persons, had lived luxuriously. The case had been skilfully treated for some months, with the usual remedies, by the gentleman's family physician—a pupil of the writer—but with only palliative results. When the early history of the case, as well as the treatment to which it had been subjected, had been explained—and this was opportunely done just as the hour had arrived for decanting the urine—a catheter was carefully introduced into the bladder by the writer, and without the slightest difficulty. The urine flowed readily, but with a feeble stream, and without any perceptible force or pain. The peculiarity of the stream, as the urine was escaping through the instrument, induced the belief that a want of contractile power of the vesical walls constituted the chief difficulty in this case, although more or less enlargement of the inferior lobe of the prostate, as well as of the gland generally, must have coëxisted also, judging from the age of the patient. An infusion of common table tea, as an injection into the bladder, suggested itself as a remedy most likely to meet the indications, while the urine was yet flowing. The tea was employed, and a single injection of four ounces of the infusion, of the ordinary strength for table use, relieved the retention, and probably it was the first time tea was ever thus used.

In this case, considerable irritation resulted; not, however, of a painful character, but rather of the nature of strangury, the desire to urinate being more urgent as well as frequent, with slight tenesmus. The irritation gradually subsided after two or three days, and the retention likewise. As evidence of the benefit derived from the tea, the writer will state that an additional fee was enclosed to him by the grateful patient, through his family physician, some weeks after the consultation. The subject of this interesting case lived some years after the tea was used, but of what disease he died, or whether the retention returned or not, he has never been informed.

Since this first trial of the tea, as a vesical injection in retention of urine, in the case just referred to, the writer has frequently employed it in the chronic diseases of the walls of the bladder, and generally with marked benefit. He has derived most frequently, however, the greatest amount of relief from it, in retention of urine, when the result chiefly of debility, relaxation, or torpor of the vesical walls, and insensibility of the mucous lining to the action of urine, with moderate enlargement of the prostate. When enlargement of the inferior lobe of the prostate gland is the chief cause of retention of urine, little, if any, benefit need be expected from the tea, unless such enlargement be complicated with œdema of the gland, or great relaxation of the vesical walls. In cystorrhœa, of subacute character and of long standing, it is a valuable topical remedy, used after intervals of eight or ten days. Occasionally, it will be found useful in incontinence of urine dependent upon relaxation of the mucous lining of the bladder, often met with in children, and young females near the age of puberty. The injection should be of the strength of tea as commonly used at the table, and may be employed in quantities varying from two to four ounces. It should be about lukewarm when introduced, and be retained from three to ten minutes. For the purpose of introducing it, a common number six or eight gum-elastic catheter, adapted with a cork to a four-ounce caoutchouc bag, will answer very well. The vesical cavity should always be evacuated of its urine and washed out with tepid water before using the tea. Should the tea cause severe irritation of the bladder, cold or tepid injections of rice-water, elm-bark, flax-seed, or the tea of althæa, may be introduced into it from time to time until it moderates, beginning not sooner than six or eight hours after the tea is discharged. Even cold water may be employed now and then, if internal heat is complained of. Should these measures fail to relieve after a sufficient time, the bowels must be freely purged. Mild demulcent and diuretic drinks will be proper, and can be beneficially used in most cases as long as the remedial irritation continues. If violent pain follows, and the patient cannot sleep of nights, a laudanum or morphine injection of small bulk should be used.—*Boston Medical and Surgical Journal.*

*April 16, 1867.*

